# High precision half-life measurement of the extinct radio-lanthanide Dysprosium-154

**DOI:** 10.1038/s41598-022-12684-6

**Published:** 2022-05-28

**Authors:** Nadine Mariel Chiera, Rugard Dressler, Peter Sprung, Zeynep Talip, Dorothea Schumann

**Affiliations:** 1 Laboratory of Radiochemistry, Paul Scherrer Institut, Villigen, Switzerland; 2 Department Hot Laboratory, Paul Scherrer Institut, 5232 Villigen, Switzerland; 3 Center for Radiopharmaceutical Sciences ETH-PSI-USZ, Paul Scherrer Institut, Villigen, Switzerland

**Keywords:** Nuclear chemistry, Nuclear physics

## Abstract

Sixty years after the discovery of ^154^Dy, the half-life of this pure alpha-emitter was re-measured. ^154^Dy was radiochemically separated from proton-irradiated tantalum samples. Sector field- and multicollector-inductively coupled plasma mass spectrometry were used to determine the amount of ^154^Dy retrieved. The disintegration rate of the radio-lanthanide was measured by means of α-spectrometry. The half-life value was determined as (1.40 ± 0.08)∙10^6^ y, with an uncertainty reduced by a factor of ~ 10 compared to the currently adopted value of (3.0 ± 1.5)∙10^6^ y. This precise half-life value is useful for the the correct testing and evaluation of p-process nucleosynthetic models using ^154^Dy as a seed nucleus or as a reaction product, as well as for the safe disposal of irradiated target material from accelerator driven facilities. As a first application of the half-life value determined in this work, the excitation functions for the production of ^154^Dy in proton-irradiated Ta, Pb, and W targets were re-evaluated, which are now in agreement with theoretical calculations.

## Introduction

Extinct radionuclides play an essential role in the description and in the reconstruction of recent galactic events, providing as well essential timescale constraints^1–4^. Even though such radionuclides became extinct soon after the formation of the Solar System, it is possible from the analysis of the abundances of their stable decay products to reconstruct the nucleosynthetic processes that occurred millions (or even billions) of years ago, e.g. in^5–9^. Over the past two decades, there has been a strong effort in the description of the mechanism responsible for the synthesis of p-nuclei. The latter is a group of proton-rich isotopes between ^74^Se and ^196^Hg that cannot be produced by either slow neutron-capture (s-process) or rapid neutron-capture (r-process) reactions^10,11^. Instead, they are produced in a network of photodisintegration and charged particle reactions called p-process^12^. Interestingly, the extinct ^154^Dy (I_α_ = 100% at E_α_ = 2.87 MeV)^13^ is one of the nuclides involved in such p-process reaction networks. Through the nuclear reactions ^154^Dy(α, γ)^158^Er^14^, ^154^Dy(γ, α)^150^Gd, and ^154^Dy(γ, 2n)^152^Dy^15^, this exotic radionuclide contributes to the synthesis of ^142^Nd, ^146^Sm, ^150^Gd, ^152^Gd, and ^158^Dy – all nuclides shielded from s- and r-nucleosynthetic processes by their respective isobars. As stated in^16,17^, knowledge on the reaction rates of nuclear reactions such as ^154^Dy(α, γ) and ^154^Dy(n,γ), respectively, is useful to test and improve the models used in the prediction of astrophysical p-process reaction rates. These models^18,19^, that correlate the observed isotopic abundances to specific nuclear reaction paths, depend on the cross section reactions, which in turn strongly depend on the half-lives of all the nuclides involved. Furthermore, due to the pure α-decay series of ^154^Dy (i.e., ^154^Dy → ^150^Gd → ^146^Sm → ^142^Nd), nucleosynthesis computations aimed to explain the processes that led to the natural isotopic abundances of ^146^Sm and ^142^Nd need to take into account the contribution of the decay of its α-unstable progenitors. Therefore, a precise half-life value of ^154^Dy is required as an input parameter^20,21^.

Besides significant applications in cosmo and geoscience, a precise value for the half-life of ^154^Dy is also required for safety evaluation of spallation target facilities, such as Accelerator Driven Systems and/or Spallation Neutron Source Facilities, that utilize targets made of heavy metals – e.g., lead, tantalum, tungsten^22^. During the spallation process, exotic α-emitting radionuclides like ^154^Dy are produced^23^. Recent theoretical calculations^24^ estimated the contribution of ^154^Dy, ^146^Sm, and ^148^Gd to the overall radiotoxicity of spallation targets to be similar to that of Po radionuclides. In that work, uncertainties on the adopted half-life values were not taken into account. Therefore, precise nuclear data for the production and decay of ^154^Dy are thus needed in order to estimate operational mode and maintenance of such facilities, as well for the decommissioning of the irradiated material.

Despite its relevance, since the discovery of ^154^Dy in 1961^25^ an accurate half-life value for this exotic radionuclide is still not available. Instead, a plethora of half-life values can be found in literature, summarized in Table 1.

**Table 1:** Half-life values for ^154^Dy available from literature.

Year	Half-life (My)	Comments	Reference
1961	1.5 ± 0.9	^154^Gd(α,4n)^154^Dy @48 MeV α-particles, number of ^154^Dy atoms estimated assuming cross section of 1 barn	^25^
1965	2.9 ± 1.5	Nuclear structure derived from α-spectrum	^26^
1971	4	Study based on the enhanced α-decay of ^154m^Dy	^27^
1985	3.0 ± 1.5	Revision of previous works with updated nuclear data	^28^
1991	1.2	Theoretical calculation	^29^

The nowadays-accepted half-life value for ^154^Dy, i.e., t_1/2_ = 3.0 My is associated with a 50% uncertainty and derives from the revision made in^28^ of the works of MacFarlane^25^ and Gōnō^27^. The half-life value in^29^ was calculated by applying a cluster model for the ground state α-decay of even-even nuclei. In most cases, little is known about the experimental procedure or about the effects taken into account in the calculation of the relative uncertainties, rendering a re-evaluation of the mentioned works difficult. It is important to mention that ^154^Dy is an extinct nuclide that can be obtained only from nuclear fusion reactions, as a by-product of spallation reactions, or by the reprocessing of nuclear waste. Therefore, the limited availability of suitable sample material, together with inherent complications in performing accurate activity measurements with long-lived nuclides, represent the main reasons for such a lack of reliable nuclear data. To overcome those difficulties, the initiative “Exotic Radionuclides from Accelerator Waste for Science and Technology – ERAWAST” was launched in 2006 at Paul Scherrer Institute (PSI)^30,31^. This long-term project aims, among others, to improve the existing nuclear databases, with a special focus on the re-determination of uncertain decay data. For this purpose, the necessary exotic radionuclides are obtained by reprocessing radioactive waste already available at the PSI site. In this work, we report on a high-precision half-life measurement of ^154^Dy, performed in the framework of ERAWAST. ^154^Dy material was obtained by reprocessing Ta samples irradiated with protons and spallation neutrons during the SINQ Target Irradiation Program (STIP) at PSI^32^. For the estimation of half-lives in the order of millions of years, we applied the so-called *direct method*. This consists of the determination of the number of radioactive atoms in a specific sample, combined with the measurement of its radioactivity. Here, the number of atoms of ^154^Dy was determined using sector field inductively-coupled plasma mass spectrometry (SF-ICP-MS) in combination with multicollector inductively-coupled plasma mass spectrometry (MC-ICP-MS). The radioactivity of ^154^Dy in the sample was measured by means of α-spectroscopy. Thin and homogeneous radioactive sources, necessary to obtain high-resolution α-spectra, were prepared using the molecular plating technique—also referred to as electrodeposition^33,34^. Following the *Guide to the expression of uncertainty in measurement – GUM* recommendations^35,36^, a realistic and complete uncertainty budget for the measured *t*_1/2_ is given as well.

## Experimental techniques and methodology

### Separation and purification of the ^154^Dy sample

**Figure 1:**
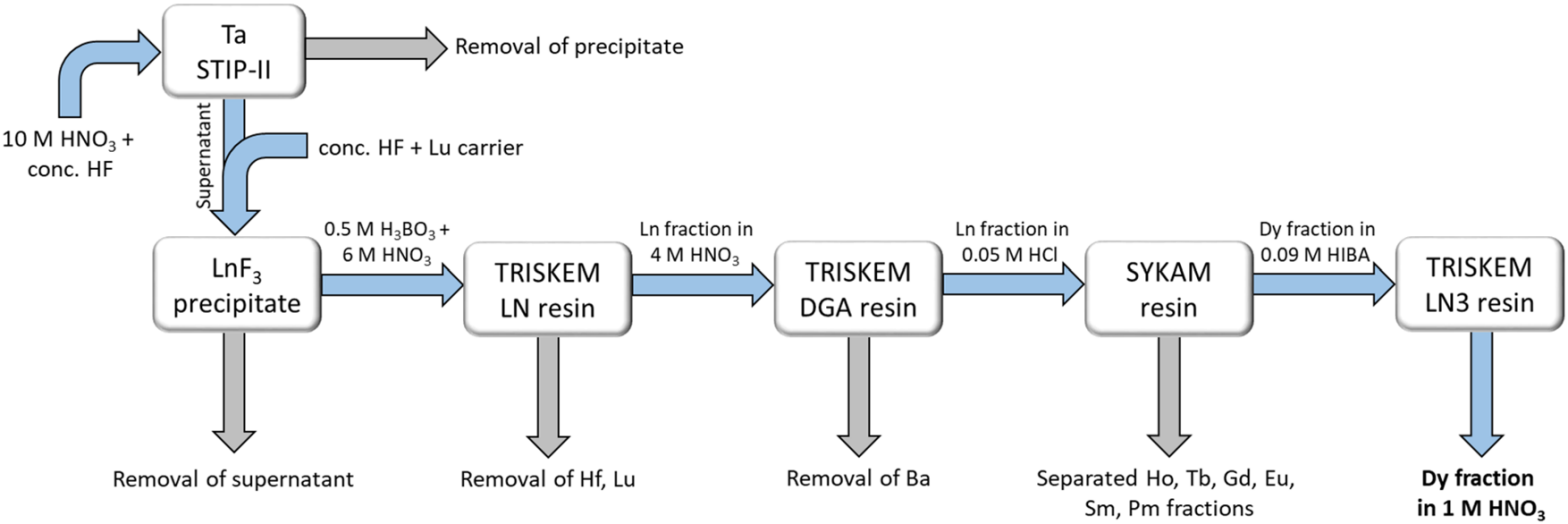
Separation scheme adopted for the retrieval of a pure Dy fraction (in 1 M HNO_3_) containing the exotic radio-lanthanide ^154^Dy. The so-obtained Dy fraction is suitable for mass spectrometric analysis without further processing. The scheme was made with the Microsoft PowerPoint 2016 software.

The Dy fraction containing ^154^Dy was obtained from the reprocessing of four Ta samples from the STIP-II project. The procedure for the dissolution of the Ta samples is described in detail in^37^. Successively, a series of ion-exchange separation processes allowed us to obtain a purified Dy fraction in 1 M HNO_3_. During the separation process, the γ-emitter ^159^Dy (t_1/2_ = (144.4 ± 0.2) d, I_γ_ = 2.29% at E_γ_ = 58 keV^38^) was added as an internal radio-tracer. The separation method for the retrieval of the Dy fraction is reported in detail in^39^. A scheme of the chemical separation steps is reported in Fig. 1.

The homogeneous Dy fraction (in 1 M HNO_3_) was collected in a scintillation vial (HDPE material, capacity: 20 ml). The total mass of the collected Dy solution – from now onwards referred to as “Dy master solution” – was determined gravimetrically (averaged value of five consecutive weightings: (5.02657 ± 0.00001) g, see Table S1). All the gravimetric steps were performed on a certified Mettler-Toledo XP56 balance (10^–6^ g scale interval), in a room with controlled temperature within 20–23 °C. Systematic uncertainties inherent to the weighing process are below 0.055%. This bias derives from the buoyancy difference between the calibration weight of the balance and the weighed solution, and therefore can be considereded negligible for differences in weight of the same solution and for mixed samples where the relative amount of each individually weighed part counts.

### Mass spectrometric analysis

The concentration of ^154^Dy in the Dy master solution was calculated from the amount of ^161^Dy (deduced by SF-ICP-MS) and from the ^154^Dy/^161^Dy isotope ratio in solution (determined by MC-ICP-MS). ^161^Dy was chosen as reference nuclide due to the absence of isobaric interferences for mass 161. All gravimetric additions were done on a Mettler-Toledo XP56 balance.

#### SF-ICP-MS measurements

SF-ICP-MS analyses were conducted using a Thermo Scientific Element 2 spectrometer, applying the medium mass resolution setting in order to minimize potential effects of molecular interferences. The plasma was operated at 1350 W. All analytes were introduced into a cyclonic PFA spray chamber using an ELEMENTAL SCIENTIFIC PFA-ST nebulizer and a peripump set, with a sample consumption of ca. 130 µl∙min^−1^. An external linear calibration was used to establish the ^161^Dy concentration in the Dy master solution. In this procedure, several dilutions of a Dy-ESI reference standard solution (Elemental scientific ^nat^Dy 10 mg∙l^-1^ ± 2% k = 2 in 2% HNO_3_, density: 1.00885 g∙ml^−1^) were repeatedly analyzed (before, in-between, and after the replicate analysis of the sample solution). In all the Dy dilutions used for the external calibration, a Re-ESI reference standard solution (Elemental scientific ^nat^Re 10 mg∙l^−1^ ± 2% k = 2 in 2% HNO_3_, density: 1.00885 g∙ml^−1^) was added as an internal reference. This allowed for cancelling potential temporal drift in instrumental signal response or plasma instability. The series of dilutions used for the external calibration scheme is presented in Tables S2–S3. An aliquot of the Dy master solution (averaged value of 5 consecutive weightings: (0.030000 ± 0.000001) g, see Table S4) was used for mass-spectrometry analysis. To this Dy aliquot, the same Re-ESI standard solution used in the preparation of calibration standards were added as an internal reference. The Dy aliquot was then diluted with a 0.28 M HNO_3_ solution, to a total weight of (13.909720 ± 0.000005) g (averaged value of 5 consecutive weightings – see Table S4). Instrumental background signals (including potential imperfect washout between analytes) were subtracted by repeated analysis of the same acid used to prepare the external standards and the sample analytes. Each of these “blank” measurements preceded the standard and the sample analyses. The ^161^Dy content in the Dy master solution was obtained by correlating the background-corrected and Re-normalized ^161^Dy signal to the external calibration line.

#### MC-ICP-MS measurements

Dy isotopic ratio analysis was conducted on the Nu Instruments Plasma 3 MultiCollector Inductively Coupled Plasma Mass Spectrometer (MC-ICP-MS) equipped with an inductively coupled Ar-plasma ion source, 16 Faraday cups, 3 Daly detectors, and 3 secondary electron multipliers. These instrumentational characteristics allow for the simultaneous measurement of up to 22 ion beams. Analytes were introduced into the system using an Elemental Scientific Apex HF desolvating nebulizer and a self-aspiring Elemental Scientific PFA-ST Microflow at a consumption rate of ca. 50 µl∙min^−1^. The plasma was operated at 1350 W forward power. Ion beams of masses 149 (Sm), 152 (Sm, Gd), 154–164 (Sm, Gd, Tb, Dy), 166–167 (Er), and 170 (Er, Yb) were collected simultaneously in Faraday cups connected to amplifier systems with a 10^11^ Ω resistors in their feedback loop. To assess potential isobaric interferences of Yb, mass 172 was monitored using a Daly ion counting detector. An aliquot of the Dy master solution was diluted by a factor of ca. 500 by addition of a pure 0.28 M HNO_3_ solution. To the diluted Dy aliquot, ^nat^Er was added, allowing for an empirical semi-external mass discrimination correction. Successively, six analyses of the so-prepared Dy sample were bracketed by 10 analyses of mixed Er-Dy solution standards. Each analysis consisted of 60 ten-second-long integrations of the ion beam intensities. Instrumental background signals were removed using interspersed analysis of the Dy sample and of the 0.28 M HNO_3_ solution used in the preparation of the analytes. Online recorded ^170^Er/^166^Er values of the admixed Er were used to determine the magnitude of instrumental mass discrimination during the analysis of the Dy sample.

### Preparation of the ^154^Dy α-source for activity measurements

**Figure 2:**
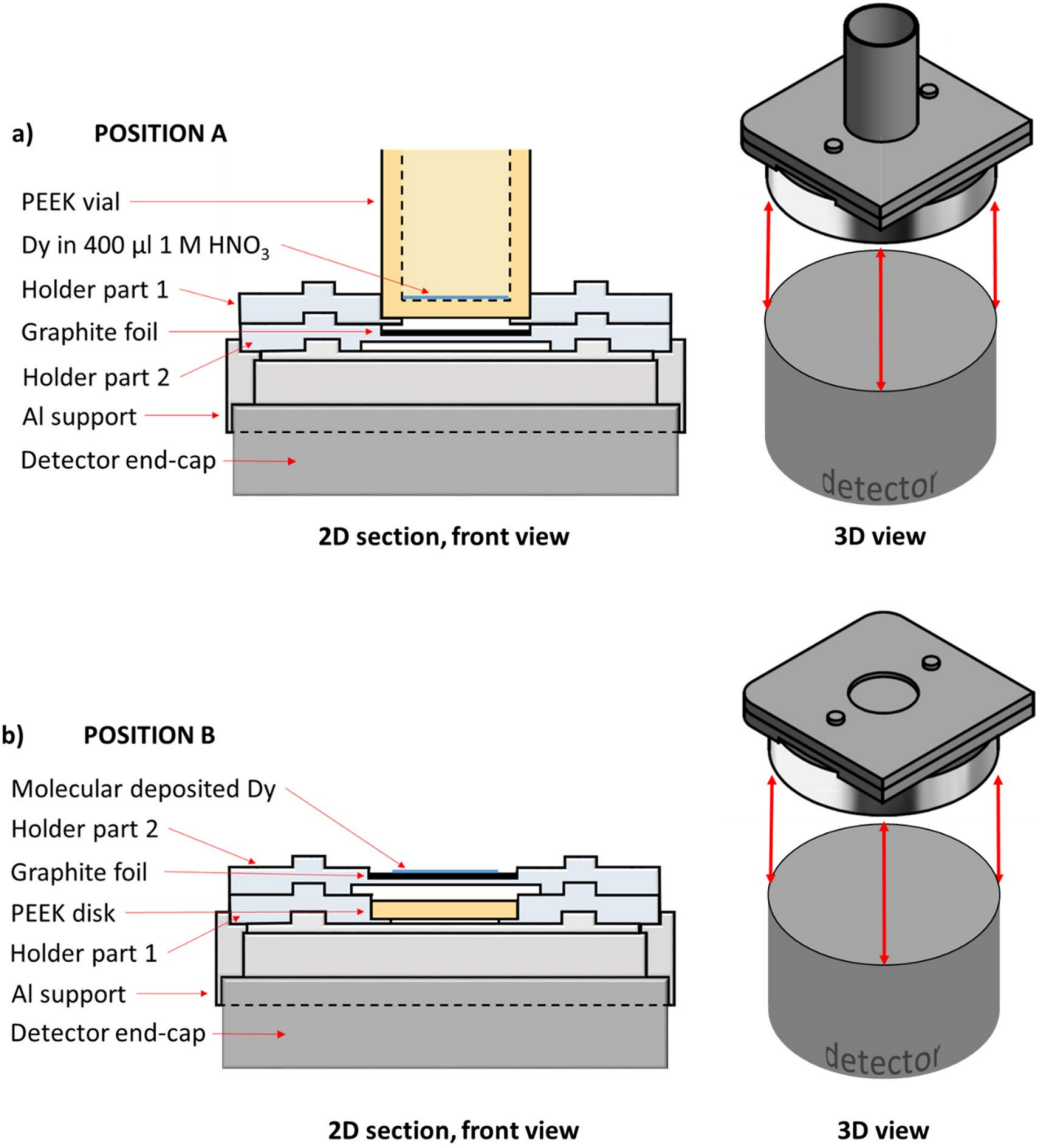
Schematic drawing of the custom-made holder, made of two interchangeable parts (namely, “Holder part 1” and “Holder part 2”), used to quantify the activity of ^159^Dy via γ-spectrometry. (**a**) 2D front section and 3D view of Position A, used to measure the γ-activity of ^159^Dy contained in the vial before molecular plating. (**b**) 2D front section and 3D view of Position B, used to quantify the γ-activity of ^159^Dy deposited on a graphite support after molecular plating. Both positions are geometrically equivalent. The acronym PEEK stands for polyether ether ketone. The drawing was made with the Microsoft PowerPoint 2016 software.

For the preparation of a thin radioactive source with the molecular plating technique, an aliquot of the Dy master solution (averaged value of 5 consecutive weightings: (2.77410 ± 0.00001) g – see Table S11) was used. The estimation of the deposition efficiency (also referred to as deposition yield) was necessary to determine the effective number of ^154^Dy atoms plated. The deposition yield was determined by monitoring the activity of the γ-tracer ^159^Dy added during the separation process (see “Separation and purification of the 154Dy sample” Section). Specifically, the activity of ^159^Dy in the Dy aliquote before molecular plating was measured, and compared to the activity of the ^159^Dy plated on the deposition foil. Since isotopes of the same element behave chemically identically, the yield of deposited ^159^Dy is thus equal to the yield of deposited ^154^Dy. For a reliable deduction of the deposition yield, both ^159^Dy γ-activity measurements had to be performed in equal geometries. This was achieved by using a custom-made holder made of two interchangeable parts (see Fig. 2), that allowed for performing γ-spectrometry measurements in two geometrically equivalent positions, namely Position A (used to quantify the activity of ^159^Dy before electrodeposition), and Position B (used to quantify the activity of ^159^Dy after electrodeposition), at a sample/detector endcap distance of 1.8 cm. Technical drawings in scale of the holder are given in the Supporting Information, Figure S2. A correction factor, that allows to convert the count rate of a volumetric sample measured in Position A to the count rate of an electrodeposited sample measured in Position B, was deduced by performing γ-spectroscopy measurements in both positions with a calibrated source of ^133^Ba (t_1/2_ = 10.54 y, I_γ_ = 32.9% at E_γ_ = 80.99 keV^40^) in 1 M HNO_3_. For Position A, a known amount of the ^133^Ba liquid source was put into a polyether ether ketone (PEEK) vial, evaporated to dryness under a N_2_ flow at T = 70 °C, and dissolved in 400 μl of 1 M HNO_3_. For Position B, a known amount of the calibrated ^133^Ba liquid source was drop-deposited onto a graphite foil (thickness: 75 μm, purity: 99.8%, Flexible Graphite, GoodFellow). The liquid was evaporated by heating the graphite foil at T = 70 °C, resulting in a point-like drop source of about 2.5 mm diameter. Further details are given in Section 2 of the Supporting Information.

All γ-spectroscopy measurements were performed with a BEGe™ (Broad Energy Germanium γ-detector, Mirion Technologies (Canberra), Inc.; crystal dimensions diameter: 61 mm, thickness: 25 mm). Data acquisition and analysis were done using the Genie™ 2000 Gamma Acquisition & Analysis Software. Energy calibration was done using a ^152^Eu (t_1/2_ = 13.53 y, I_γ_ = 28.41% at E_γ_ = 121.78 keV^41^) point-source (Physikalisch-Technische Bundesanstalt – PTB). The energy resolution FWHM (Full Width at Half Maximum) was 0.54 keV at 58 keV.

#### γ-activity measurements before molecular plating (Position A)

For the γ-spectrometry measurement in Position A, the Dy aliquot was transferred from the HDPE vial to a custom-made PEEK vial (internal diameter: 20 mm, thickness at the bottom: 1 mm), and evaporated to dryness at 70 °C under a N_2_ gas flow. To ensure a complete transfer of the Dy aliquot, the HDPE vial was rinsed with 10 ml 1 M HNO_3_, transferring the washing solution to the PEEK vial, and evaporating the liquid to dryness. This process was repeated 5 times. Then, 400 μl of 1 M HNO_3_ were added in order to dissolve the dried Dy solid. The added volume corresponded to the minimum volume that would entirely cover the bottom of the PEEK vial. This step was necessary to avoid attenuation of the γ-rays of ^159^Dy at 58 keV due to the presence of Dy(NO_3_)_3_ crystals, as well as to ensure a specific geometry equivalent to the one of the electrodeposited radioactive source. The PEEK vial containing the Dy dissolved in 1 M HNO_3_ was placed in the custom-made holder. A graphite foil (thickness: 75 μm, purity: 99.8%, Flexible Graphite, GoodFellow) was inserted between the bottom of the PEEK vial and the detector endcap, as shown in Fig. 2a. The γ-measurement of the ^159^Dy contained in the PEEK vial was performed for 540 s.

#### Molecular plating

After the γ-spectroscopy measurements, the Dy solution was transferred from the PEEK vial to a HDPE vial and evaporated to dryness at 70 °C under a N_2_ gas flow. To ensure a complete transfer of the Dy, the PEEK vial was rinsed with 5 ml 1 M HNO_3_, the washing solution was transferred to the HDPE vial, and the liquid was evaporated to dryness at 70 °C under a N_2_ flow. This process was repeated 5 times. The dried Dy was then dissolved in 6 M HNO_3_ to promote the formation of nitrate species and again evaporated to dryness at 70 °C under a N_2_ flow. Any organic species that might derive from the separation process described in “Separation and purification of the 154Dy sample” Section was digested by the addition of modified aqua regia, i.e., 1.5 ml 30% (w/w) H_2_O_2_ + 4.5 ml conc. HCl + 1.5 ml conc. HNO_3_. The solution was evaporated to dryness at 80 °C under a N_2_ flow, and the residual solid was re-dissolved in a mixture of 2 ml conc. HNO_3_, 6 ml conc. HCl, and 2 ml conc. HF for the destruction and removal of any silica compound that might derive from the ion exchange resins used in the separation of the Dy fraction from the Ta matrix. The solution was then evaporated to dryness (80 °C under a N_2_ flow), dissolved in 1 M HNO_3_, and re-evaporated to dryness (70 °C in N_2_ flow). Finally, the electroplating solution was obtained by adding a 50:50 methanol (MeOH) / isopropanol (iPrOH) mixture to the dried solid residue, for a total volume of 10 ml. The liquid was then transferred to the electrodeposition cell made of polytetrafluoroethylene (PTFE). A description of the molecular plating setup can be found in^42^. Before electrodeposition, a cleaning procedure (stepwise rinsing in 1 M HNO_3_, MilliQ water, and iPrOH) was applied to the PTFE cell and to the spiral Pt wire (anode). The cathode, made of a copper block, was cleaned with 0.1 M citric acid, washed with MilliQ water, and rinsed with iPrOH. The graphite deposition foil (thickness: 75 μm, diameter of deposition area: 20 mm, GoodFellow Cambridge Ltd.) was cleaned with iPrOH before molecular plating. For a constant deposition temperature, the setup was implemented with a Peltier cooler at the cathode, maintaining the graphite foil at 15 °C during the entire plating procedure. The distance between the two electrodes was approximately 10 mm. The electrodeposition of Dy on the graphite foil was achieved in 8 h by applying a constant voltage of 550 V.

#### γ-activity measurements after molecular plating (Position B)

The activity of the ^159^Dy contained in the Dy deposited on the graphite foil was measured by placing the foil in Position B (see Fig. 2b). In between the graphite foil and the BEGe™ detector endcap, a PEEK disk (thickness = 1.0 mm, identical to the bottom of the PEEK vial) was inserted, as shown in Fig. 2b. The γ-spectrometry measurement of the ^159^Dy deposited on the graphite foil was conducted for 2.16∙10^6^ s (i.e., 25 days).

### ^154^Dy α-activity measurement

The graphite foil with the electrodeposited Dy was then transferred to an α-chamber for the measurement of the α-activity of ^154^Dy. α-spectrometry was performed using the Alpha Analyst Integrated Alpha Spectrometer (model A-450-21AM, Canberra) equipped with a silicon semiconductor detector (Passivated Implanted Planar Silicon – PIPS. Detector sensitive area: 450 mm^2^; energy resolution FWHM: 21 keV). Data acquisition and analysis were done using the Genie™ 2000 Alpha Analysis Software. Energy calibration of the detector was performed with an α-source of ^148^Gd (t_1/2_ = 74.6 y, I_α_ = 100% at E_α_ = 3.182 MeV^43^), and a mixed ^239^Pu (t_1/2_ = 2.41 y, I_α_ = 70.77% at E_α_ = 5.157 MeV^44^), ^241^Am (t_1/2_ = 432.8 y, I_α_ = 84.8% at E_α_ = 5.486 MeV^45^), and ^244^Cm (t_1/2_ = 18.1 y, I_α_ = 76.90% at E_α_ = 5.805 MeV^46^) α-source. For all measurements, the sample-detector distance (SDD) was 10.4 mm. The efficiency calibration of the detector was performed with a certified ^241^Am standard source (PTB, calibration reference n° PTB-6.11-2016-1769, A = (539 ± 11) Bq @01.11.2016 00:00:00 MEZ, uncertainty with k = 2), having the same diameter (20 mm) as the Dy deposition area on the graphite foil. Geometrical differences between the ^241^Am standard source and the Dy electrodeposited sample were further minimized by using holders with the same SDD for both samples. The activity of the ^154^Dy electrodeposited sample was measured at a defined solid angle. The α-spectrometry measurement of the electrodeposited Dy layer was conducted for 5∙10^5^ s (i.e., 5.8 days).

## Results and discussion

### Mass spectrometry analysis

The ^161^Dy content in the Dy sample for SF-ICP-MS analysis, resulting from the average of six analyses (see Table S5), was deduced to be (0.01203 ± 0.00046) nmol∙g^-1^. In the data analysis for the external standards, the accepted natural isotope composition of Dy given in^47^ was used. Possible isobaric interferences from Sm and Gd on the ^154^Dy ion-beam were considered negligible, since the ratios of the signals ^149^Sm/^154^total (where ^154^total stands for the bulk signal on mass 154 without element distinction), ^152^(Sm,Gd)/^154^total, ^155^Gd/^154^total, and ^157^Gd/^154^total are all below 10^−3^. Therefore, a correction of the ^154^total/^161^Dy value for isobaric interferences from Sm and Gd – assuming natural isotope compositions of the interfering species – would result to a 0.02% lower value. Therefore, no isobaric correction was undertaken. Likewise, potential interferences from Yb on mass 170 were insignificant since the signal ratios ^172^Yb/^170^total (where ^170^total stands for the bulk signal on mass 170 without element distinction) were below 10^−5^. The signal ratio ^170^Yb/^170^total was in the order of 10^−6^. The relation between the exponential mass discrimination factors for Er and Dy^48^ was established from the analyses of the natural Er-Dy solution standards, considering the natural isotopic abundances reported in^47,49^ and the nuclide masses listed in^50^. This (linear) relation, together with the exponential mass discrimination factors for Er, allowed for accurate mass discrimination corrections of the ^154^Dy/^161^Dy ratio in the Dy master solution. As a final result from the average of six analyses (see Table S6), the ^154^Dy/^161^Dy ratio was determined as (0.277317 ± 0.000085). The concentration of ^154^Dy in the Dy master solution was assessed as 1.547 nmol∙g^−1^, with an uncertainty of 3.82% (k = 1). The latter includes the uncertainty on the concentration of the Dy standard used for SF-ICP-MS.

### Estimation of the molecular plating efficiency

From the comparison of the ^133^Ba measurements in Position A and Position B, a geometry conversion factor of (1.045 ± 0.015) was deduced (see Section 2 of the Supporting Information). From the knowledge of the ^154^Dy concentration in the master solution, it follows that the amount of ^154^Dy contained in the aliquot (mass: (2.77410 ± 0.00001) g, see Table S11) used for the preparation of the thin radioactive α-source corresponds to (4.291 ± 0.163) nmols. The count rate of the ^159^Dy tracer before electrodeposition contained in the PEEK vial was quantified to be (2.7870 ± 0.0232) counts∙s^−1^ @13.08.2020, whereas the count rate of the ^159^Dy tracer electrodeposited on the graphite foil amounted to (0.0047 ± 0.0002) counts∙s^−1^ @30.03.2021. Parameters used for the estimation of the count rates are given in Table 2. The corresponding γ-spectra are included in Section 2 of the Supporting Information. Considering the decay of ^159^Dy in the time elapsed between the measurement in Position A and the measurement in Position B (i.e., 229.16 days), and the geometry conversion factor, a deposition yield of (0.53 ± 0.02)% was calculated. The reported deposition yield includes the 0.14% uncertainty on the half-life of the ^159^Dy tracer^38^. Taking into account the uncertainties deriving from (SF-MC)-ICP-MS and from the ^159^Dy activity measurements, (0.0226 ± 0.0009) nmols of ^154^Dy were electrodeposited on the graphite foil, equivalent to (1.361 ± 0.052)∙10^13^ atoms of ^154^Dy.

**Table 2:** Count rate (in counts∙s^-1^) at 58 keV corresponding to the γ-measurements of ^159^Dy in Position A and Position B.

	Date (dd.mm.yyyy)	t_real_ (s)	t_life_ (s)	Count rate (counts∙s^-1^)
Position A	13.08.2020	5400	5381	2.787 ± 0.023
Position B	30.03.2021	2,160,000	2,158,961	0.0047 ± 0.0002

The exact dates (in dd.mm.yyyy format) at which the measurements were performed are given. The real time (t_real_, in seconds) and life time (t_life_, in seconds) of each measurement are reported as well.

### ^154^Dy α-activity measurement

**Figure 3:**
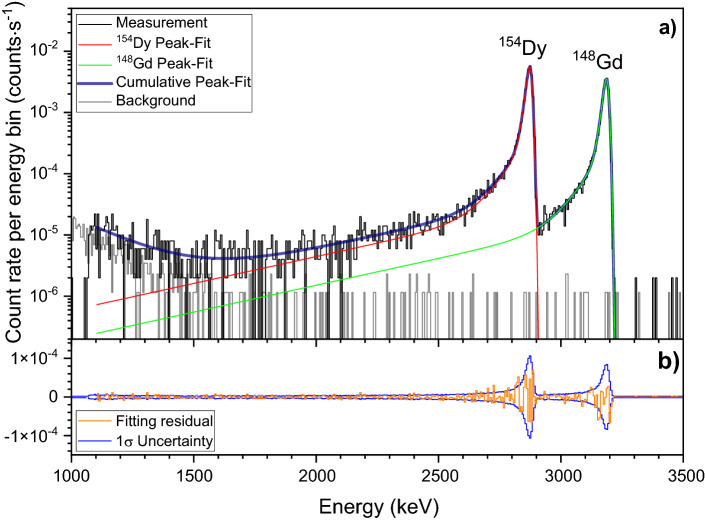
(**a**) α-spectra (black line) of the electrodeposited Dy on a graphite foil (SDD = 10.4 mm, counting time = 5∙10^5^ s or 5.8 days). The histogram bin size is 5.925 keV. The presence of ^148^Gd, contained in traces in the Dy separated fraction (isotope ratio ^154^Dy:^148^Gd ≈ 28,000:1), is clearly visible. The model (navy line) that comprises the fit of the ^154^Dy peak (red line), the ^148^Gd peak (green line), as well as the electronic low energy noise, is shown. In addition, the background (light grey line) recorded during a period of 10 days (8.64∙10^5^ s) is superimposed. (**b**) Fitting residuals. The residuals (orange line) are consistent with the Poisson counting statistics of the spectrum (1σ uncertainty, blue line).

As shown in Fig. 3, a slight contamination of ^148^Gd was visible in the α-spectrum. Thus, a deconvolution method based on a combination of^51^ and^52^ for a precise peak-shape fitting in the (1.1–3.5) MeV energy range (see Section 3 of the Supporting Information for further details), was applied. The fitting residuals, given as well in Fig. 3, are consistent with the measured counting statistics. The activities of ^154^Dy and ^148^Gd, together with the one of the ^241^Am standard source used for efficiency calibration, are given in Table 3.

**Table 3:** Activity (A, in Bq) of the ^154^Dy electrodeposited on the graphite foil, together with the activity of the ^148^Gd impurity.

	t_real_ (s)	t_life_ (s)	Energy range (MeV)	Count rate (counts∙s^-1^)	A (Bq)
^241^Am	500	501	5.3–5.6	78.84 ± 0.56	534.9 ± 5.5^a^
^154^Dy	500,000	500,001	1.1–3.5	0.03133 ± 0.00044	0.2126 ± 0.0040
^148^Gd	500,000	500,001	1.1–3.5	0.02006 ± 0.00032	0.1361 ± 0.0027

^a^Activity of the source at the date of the efficiency calibration (20.07.2021), calculated using (432.6 ± 0.6) y as the half-life of ^241^Am, as reported in ^45^.

The activity of the ^241^Am standard source used for efficiency calibration is indicated as well. For each measurement, the real time (t_real_, in seconds) and the life time (t_life_, in seconds) is reported. The energy range considered for the calculation of the count rate (in counts∙s^-1^) of each α-peak is given as well.

### ^154^Dy half-life

A reliable half-life value is obtained by applying the following equation:

1 $$ t_{1/2} = \ln 2 \cdot \frac{N}{A} $$

where *N* is the number of atoms of ^154^Dy—i.e., (1.361 ± 0.052)∙10^13^, and *A* is their activity—i.e., (0.2126 ± 0.0040) Bq. Here, the half-life value for ^154^Dy was determined as (1.40 ± 0.08) My, with an estimated total uncertainty of 5.6%. All the uncertainties (see the uncertainty budget in Table 4) were combined under the assumption that they are completely uncorrelated. For the half-life calculation, 1 year was considered to be equal to 365.242198 days.

**Table 4:** Uncertainties budget (with k = 1) for the ^154^Dy half-life measurement.

Main parameter	Source of uncertainty	Partial contribution	Combined Uncertainty (%)
^**154**^**Dy in master solution**			3.81%
*Dilution factor*		0.012%	
^*154*^*Dy/*^*161*^*Dy isotope ratio*		0.014%	
^*161*^*Dy concentration*		3.54%	
*External calibration curve*		1.02%	
*Dy-ESI standard*		1.0%	
**Weighting**			0.055%
**Deposition yield**			3.70%
*Yield, not corrected*		3.42%	
*Correction factor*		1.42%	
*t*_*1/2*_^*133*^*Ba*		0.057%	
*t*_*1/2*_^*159*^*Dy*		0.14%	
^**154**^**Dy activity**			1.89%
^*154*^*Dy α-counting*		1.42%	
^*241*^*Am α-counting*		0.71%	
^*241*^*Am ref. standard*		1.02%	
*t*_*1/2*_^*241*^*Am*		0.14%	
**Total**			5.6%

### Re-evaluation of the production cross-sections of ^154^Dy from proton irradiated Pb, Ta, and W targets

**Figure 4:**
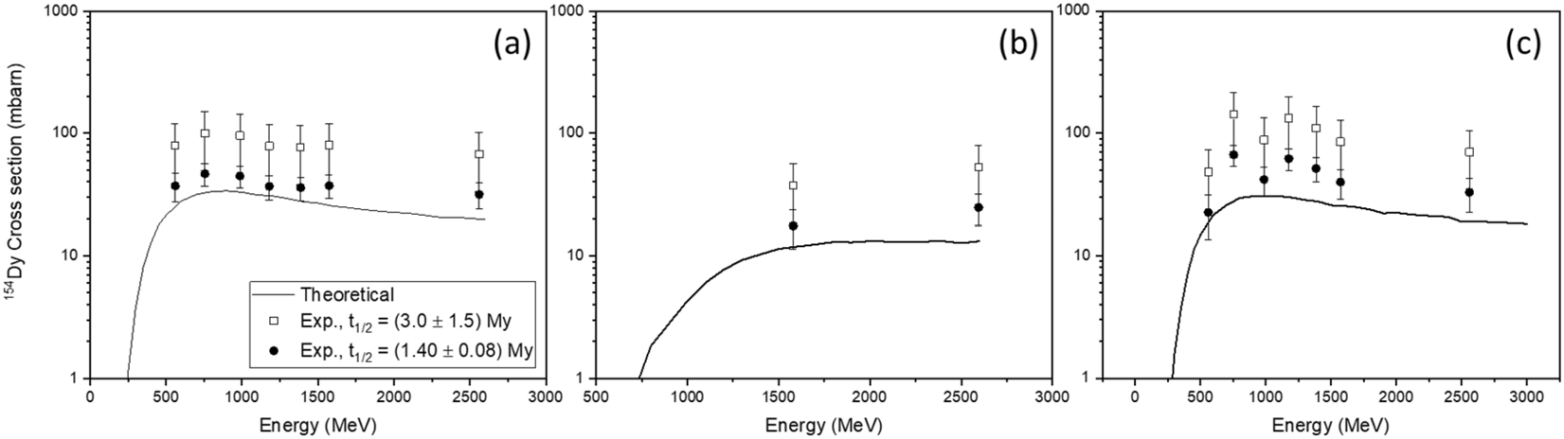
Excitation functions for the production of ^154^Dy in proton irradiated Ta (**a**), Pb (**b**), and W (**c**) targets. Solid lines: theoretical values obtained with INCL +  + and ABLA 07 codes; filled dots: experimental values using t_1/2_ = (1.40 ± 0.08) My; open squares: experimental values using t_1/2_ = (3.0 ± 1.5) My.

The production cross-sections of ^154^Dy in proton-irradiated Ta, Pb, and W targets previously reported in^37,53,54^ were re-evaluated applying Eq. 2:

2 $$ \sigma^{*} = \sigma \cdot \frac{{t_{1/2}^{*} }}{{t_{1/2} }} $$

where σ* is the re-calculated cross section, *σ* is the experimental cross section from literature, t^*^_1/2_ is the half-life value of ^154^Dy determined in this work, and *t*_*1/2*_ is the currently adopted half-life value for ^154^Dy. As shown in Fig. 4, a significant decrease in the uncertainty of the experimental excitation functions for ^154^Dy was achieved, with the new results being in agreement with theoretical calculations obtained using INCL +  + and ABLA 07 codes^55–57^. For the sake of completeness, the comparison with the cross-section results derived using t_1/2_ = (3.0 ± 1.5) My is depicted as well.

## Conclusions

By applying state-of-the-art radiochemical separation techniques, in conjunction with high precision activity measurements, the half-life of the pure α-emitting radio-lanthanide ^154^Dy was determined as (1.40 ± 0.08) My. This result is in very good agreement with the half-life value deduced by MacFarlane et al.^25^. Compared to the currently adopted value of (3.0 ± 1.5) My, the new measurement drastically reduced the uncertainty by a factor of ~ 10. The increased accuracy of the half-life of ^154^Dy can be exploited in, e.g., the development of a novel dating tool that uses the ^154^Dy/^142^Nd radiometric pair as chronometer. As a first application of the updated decay data for ^154^Dy, the experimental cross sections for the Ta(p,x)^154^Dy, Pb(p,x)^154^Dy, and W(p,x)^154^Dy nuclear reactions were re-evaluated. The resulting cross-sections are now in better agreement with the theoretical values obtained with INCL +  + and ABLA 07 codes. Since the shorter half-life value measured in this work implies a greater decay rate for ^154^Dy at a given time *t*, a re-evaluation of the toxicity of spallation targets that contain ^154^Dy as by-product is required.

For future calculations or derivations using the half-life of ^154^Dy, it is recommended to adopt the value deduced in this work.

## Supplementary Information

Supplementary Information.

## Data Availability

All relevant data are within the paper and its Supporting Information file. The datasets generated during and/or analysed during the current study are available from the corresponding author on reasonable request.
