# Supplementary material for: High precision half-life measurement of the extinct radio-lanthanide Dysprosium-154
Source: Sci Rep. 2022 May 28;12:8988. doi: 10.1038/s41598-022-12684-6 (PMC9148308; doi:10.1038/s41598-022-12684-6)
Supplement: Supplementary file 1 — Supplementary Information. [file 41598_2022_12684_MOESM1_ESM.docx]

**SUPPORTING INFORMATION**

**High precision half‑life measurement of the extinct radio‑lanthanide Dysprosium-154**

Nadine Mariel Chiera^1,*^, Rugard Dressler^1^, Peter Sprung^2^, Zeynep Talip^1,3^, Dorothea Schumann^1^

^1^ Laboratory of Radiochemistry, Paul Scherrer Institut, Villigen PsSI, Switzerland.

^2^ Department Hot Laboratory, Paul Scherrer Institut, 5232 Villigen PSI, Switzerland.

^3^ Center for Radiopharmaceutical Sciences ETH-PSI-USZ, Paul Scherrer Institut, Villigen PSI, Switzerland.

* Corresponding author

E-mail: nadine-mariel.chiera@psi.ch

**1. Mass spectrometric analysis**

***1.1 Total amount of retrieved Dy***

**Table S1.** Total amount of Dy master solution, in grams, calculated as the difference between gross weight (Dy solution + HDPE vial) and tare (HDPE vial). The mean and standard deviation (SD) of five consecutive weightings is indicated.

| **Weighting**  **#** | **Vial**  **(tare, g)** | **Dy solution + vial (gross weight, g)** | **Dy solution**  **(net weight, g)** |
| --- | --- | --- | --- |
| **1** | 6.79404 | 11.82061 | 5.02657 |
| **2** | 6.79409 | 11.82061 | 5.02652 |
| **3** | 6.79402 | 11.82062 | 5.02660 |
| **4** | 6.79403 | 11.82063 | 5.02660 |
| **5** | 6.79403 | 11.82062 | 5.02659 |
| **Mean** | 6.79404 | 11.82062 | 5.02657 |
| **SD** | 2.8∙10^-5^ | 7.1∙10^-5^ | 3.4∙10^-5^ |

***1.2 Calibration curve for SF-ICP-MS analysis***

**Table S2.** Dilution series (50, 10, 1, 0.5, 0.1, 0.05 ppb) derived from a Dy-ESI reference standard (Elemental scientific ^nat^Dy 10 mg∙l^-1^ ± 2% k = 2 in 2% HNO_3_) and a Re-ESI reference standard (Elemental scientific ^nat^Re 10 mg∙l^-1^ ± 2% k = 2 in 2% HNO_3_) were used for the external standard calibration scheme. From the Dy-ESI standard, a 50 ppb solution was prepared and used for the calibrations at 50, 10, 1 and 0.5 ppb. Then, the 1 ppb Dy solution was used for preparing the dilutions at 0.1 ppb and 0.05 ppb. Aliquots of Re-ESI standard were added as an internal reference. All solutions used for the external calibration as well as the blank solution were prepared using the same 0.28 M HNO_3_.

| **Sample**  **conc.**  **(ppb)** | **Standards added (g)** | | | | **Mass fraction (µg∙g^-1^)** | |
| --- | --- | --- | --- | --- | --- | --- |
|  | **Dy-ESI**  **50 ppb dilution** | **Dy-ESI**  **1 ppb dilution** | **Re ESI standard** | **0.28 M HNO_3_** | **Dy-ESI** | **Re-ESI** |
| **50** | 14.11823 | **---** | 0.05020 | **---** | 5017.13 ± 0.25 | 3543.09 ± 0.35 |
| **10** | 2.80821 | **---** | 0.05029 | 11.24042 | 1002.86 ± 0.09 | 3566.94 ± 0.35 |
| **1** | 0.27954 | **---** | 0.05056 | 13.61587 | 100.92 ± 0.01 | 3625.42 ± 0.36 |
| **0.5** | 0.14248 | **---** | 0.05035 | 13.73841 | 51.50 ± 0.01 | 3614.18 ± 0.38 |
| **0.1** | **---** | 1.41610 | 0.05022 | 12.53421 | 10.21 ± 0.00 | 3587.01 ± 0.36 |
| **0.05** | **---** | 0.70645 | 0.05044 | 13.23594 | 5.09 ± 0.00 | 3604.70 ± 0.36 |
| **blank** | **---** | **---** | 0.05007 | 14.01311 | **---** | 3560.36 ± 0.35 |

The SF-ICP-MS individual measurements of all calibration solutions, of the diluted ^154^Dy sample and of the blank solution were performed as 40 times sweep over the mass range from 137 to 187. The ions reaching the detector for each mass were counted for 240 ms in each sweep with the 20% highest area of the signal peak (equivalent to 48 ms of observation per sweep) forming the basis of the recorded count-rate. The two most extreme results of these sweeps were discarded as outliers. With the remaining 38 sweeps, an averaged count-rate was calculated. For each sample, 6 individual measurements were performed and used for data analysis, with exception of the 0.1 ppb solution where one entire measurement was discarded due to technical problems during the data acquisition. The ratio of the recorded ions counts per second (CPS) of ^161^Dy and of ^187^Re (the later normalized to the mass-fraction of the Re-ESI added in the solutions), were calculated to suppress changes of the ion flux. In Table S3 the average values over all the calibration solutions and the blank is given. A weighted linear fit was used to determine the slope (β) and offset (α) of the external calibration according to the equation:

$\mathcal{w=R\cdot}\beta-\alpha$ (S1)

Where $\mathcal{R}$ denotes the averaged and normalized isotope ratios, i.e. the ratio of the recorded ions counts per second of ^161^Dy and of ^187^Re (with the latter being normalized by the mass-fraction of the Re-ESI in the calibration solutions), and $\mathcal{w}$ the mass fraction of the Dy-ESI standard in the calibration solutions. Values of $\mathcal{R}$ are given in Table S3. In addition, the Studentized residuals of a linear fit using all data points (corresponding to the red dashed-dot line in Figure S1) are given, indicating the measurement value of the 50 ppb sample as an outlier.

**Table S3.** Results of SF-ICP-MS measurements used to establish a linear external calibration.

| **Sample conc.**  **(ppb)** | **Mass-fraction**  **(µg∙g-1)** | **Dy-ESI**  **(µg∙g-1)** | **Averaged and normalized isotope ratios** $\mathcal{R}$  **(µg)** | | | **Studentized fit-residuals** |
| --- | --- | --- | --- | --- | --- | --- |
|  |  |  | average | uncert. | rel. uncert. |  |
| **0.05** | 5.09528 | 0.00074 | 1.59 | 0.14 | 8.6% | 0.0137 |
| **0.1** | 10.2080 | 0.0014 | 2.77 | 0.13 | 4.7% | -0.1123 |
| **0.5** | 51.4945 | 0.0059 | 14.29 | 0.51 | 3.6% | 0.1402 |
| **1** | 100.923 | 0.011 | 26.49 | 0.55 | 2.1% | -0.5787 |
| **10** | 1002.861 | 0.094 | 266.50 | 2.20 | 0.8% | -1.9885 |
| **50** | 5017.13 | 0.25 | 1392 | 14 | 1.0% | 125.4798 |
| **blank** | 0 | 0 | 0.210 | 0.031 | 15.0% | 0.0075 |

The linear fit excluding the data corresponding to the 50 ppb sample reveals for the slope a value of β = (3.766 ± 0.038) g^-1^ (with an uncertainty contribution of 0.024 g^-1^ from the fit procedure and 0.030 g^-1^ from the propagation of the data uncertainties), and for the offset α = (0.7659 ± 0.0096) µg∙g^-1^ (with an uncertainty contribution of 0.0096 µg∙g^-1^ from the fit procedure and 0.0002 µg∙g^-1^ from the propagation of the data uncertainties). The standardized regression coefficient of the fit is 0.99984. This linear regression fit, together with the 68% confidence band, is shown in Figure S1 in green.

**
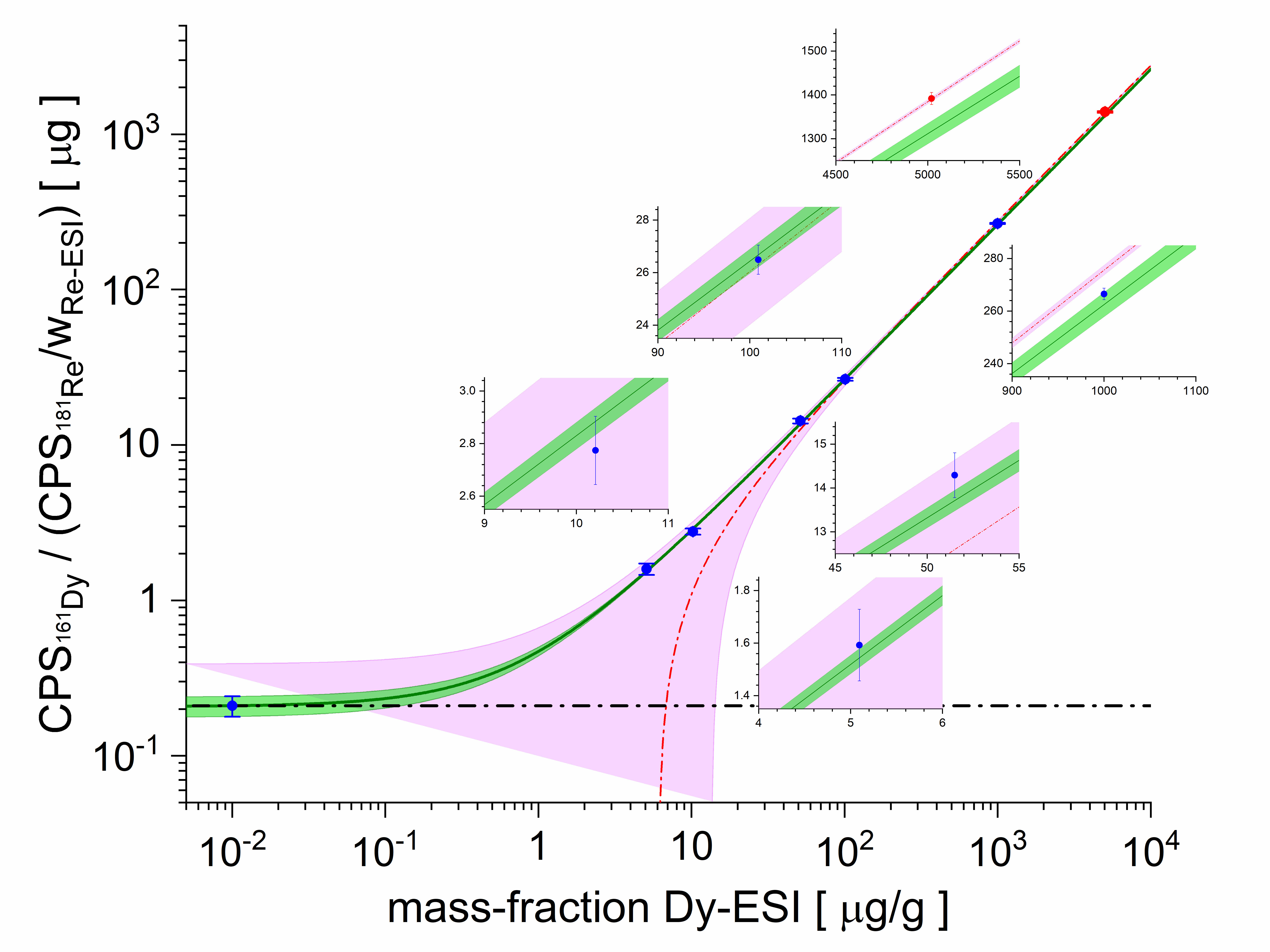
**

**Figure S1.** Linear regression of the averaged and normalized isotope ratios versus the mass fraction of the Dy-ESI standard in the solutions. The black dashed-dotted line indicates the measured blank level. Displayed are the measurement results from Table S3 (blue dots), together with the result of a weighted last square linear fit (green line) in a double logarithmic plot. The data point obtained by analyzing the 50 ppb sample is indicated with a red dot. Due to graphical reasons, the blank value was displayed at a mass-fraction of 0.01 µg∙g^‑1^, however for the fitting procedure the correct value of 0 µg∙g^-1^ was used. The obtained blank level is indicated by the horizontal black dashed-dot line. The green shaded area indicates the 68% confidence region of the linear fit excluding the measurement of the 50 ppb sample. The pink shaded area refers to the 68% confidence region of the fitting using all the data points (i.e., including the measurement of the 50 ppb sample). Zoomed regions around the measurement data points are included as insets.

The SF-ICP-MS analyses resulted in an averaged and normalized isotope ratio $\mathcal{R}$ of (277.50 ± 0.98) μg. The analyzed aliquot of Dy master solution (see Table S4 for its gravimetrically determined amount) contains therefore a content of (0.01203 ± 0.00046) nmol of ^161^Dy per gram of solution. The aliquot of Dy master solution used for ICP-MS analysis was diluted by a factor of (463.596 ± 0.055). Hence, the Dy master solution contains (1.547 ± 0.059) nmol of ^161^Dy per gram of solution, equivalent to (93.2 ± 3.6)∙10^13^ atoms per gram of solution.

**Table S4.** Gravimetrically determined amount (in grams) of the aliquot of Dy master solution used for mass-spectroscopy analysis. To the aliquot, Re ESI standard solution was added as internal reference. The sample was then diluted with 0.28 M HNO_3_. The mean and standard deviation (SD) of five consecutive weightings is indicated.

| **Weighting**  **#** | **Dy aliquot**  **(g)** | **Re ESI standard (g)** | **0.28 M HNO3**  **(g)** | **Total**  **(g)** |
| --- | --- | --- | --- | --- |
| **1** | 0.03000 | 0.04996 | 13.82975 | 13.90971 |
| **2** | 0.03000 | 0.04996 | 13.82976 | 13.90972 |
| **3** | 0.03001 | 0.04996 | 13.82977 | 13.90974 |
| **4** | 0.03001 | 0.04995 | 13.82976 | 13.90972 |
| **5** | 0.03000 | 0.04997 | 13.82976 | 13.90973 |
| **Mean** | 0.03000 | 0.04996 | 13.82975 | 13.90972 |
| **SD** | 2.5∙10^-6^ | 7.1∙10^-6^ | 7.1∙10^-6^ | 1.1∙10^-5^ |

***1.3 (SF/MC)-ICP-MS results***

**Table S5.** Concentration (in nmol∙g^-1^) of ^161^Dy in the Dy sample for SF-ICP-MS analysis, determined by 6 consecutive mesurements of a Dy analyte composed of 0.03000 g of Dy master solution + 0.04996 g of Re ESI + 13.82975 g of 0.28 M HNO_3_.

| **Analysis #** | **^161^Dy concentration** |
| --- | --- |
| **1** | 0.01152 ± 0.00044 |
| **2** | 0.01229 ± 0.00047 |
| **3** | 0.01163 ± 0.00045 |
| **4** | 0.01220 ± 0.00047 |
| **5** | 0.01248 ± 0.00048 |
| **6** | 0.01208 ± 0.00046 |
| **Mean** | 0.01203 ± 0.00046 |

**Table S6.** ^154^Dy/^161^Dy ratio in the Dy master solution, determined by MC-ICP-MS. “internal SD” refers to the standard deviation of the 60 ten-second-long signal integrations per each sample analysis. In addition to the values of the individual measurements the mean value, the standard deviation and the uncertainty propagation of the internal standard deviation is given. This results in an averaged ^154^Dy/^161^Dy ratio of (0.277317 ± 0.000085).

| **Analysis #** | **^154^Dy/^161^Dy** | **internal SD/√N** |
| --- | --- | --- |
| **1** | 0.27735 | 0.00018 |
| **2** | 0.27726 | 0.00016 |
| **3** | 0.27719 | 0.00015 |
| **4** | 0.27731 | 0.00017 |
| **5** | 0.27730 | 0.00020 |
| **6** | 0.27749 | 0.00019 |
| **Mean** | 0.277317 |  |
| **SD** | 0.000046 | 0.000072 |

**2. γ-spectrometry measurements**

***2.1 Aluminum holder for ^159^Dy γ-measurements***

**
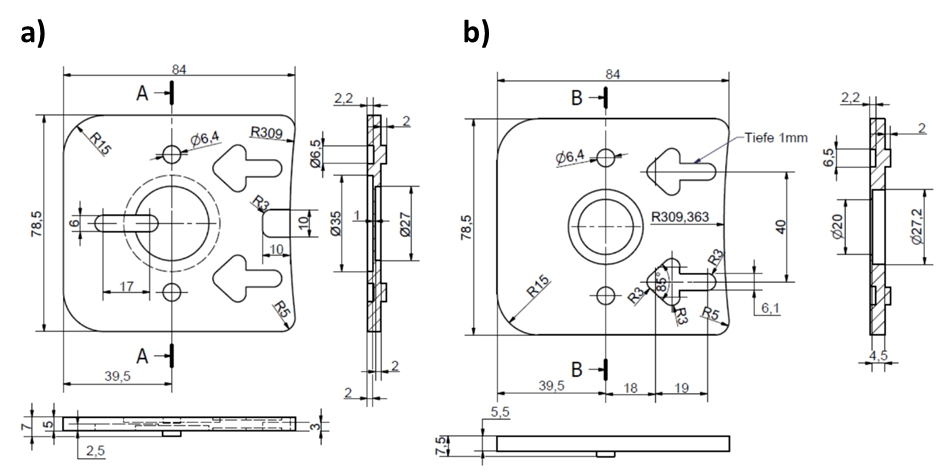
**

**Figure S2.** Schematic drawing, in scale, of the custom-made Al holder made of two interchangeable parts, namely (a) Part 1 and (b) Part 2. The technical drawing was made with the Autodesk Inventor 2019 software.

***2.2 Geometry conversion factor for Position A and Position B***

The equivalency of Position A and Position B in terms of γ-ray detection rates was checked by comparing γ-measurements in the mentioned positions using ^133^Ba (t_1/2_ = 10.539 y, I_γ_ = 32.9 % at E_γ_ = 80.99 keV) samples produced from a ^133^Ba liquid stock solution in 1 M HNO_3_. First, the specific activity of the ^133^Ba liquid stock solution was determined by comparing the activity of a ^133^Ba drop-source with the one of a certified ^133^Ba point-like reference source made at the PTB. After determining the specific activity of the ^133^Ba liquid source, measurements on the equivalency of Position A and Position B were performed. A geometry conversion factor, to be taken into account in the calculation of the deposition yield, was thus derived. Data acquisition and analysis were done using the Genie^TM^ 2000 Gamma Acquisition & Analysis Software.

**Specific activity of the ^133^Ba liquid source.** During the reprocessing of the STIP-II specimens [see reference 37], a sample of ^133^Ba in 1 M HNO_3_ (here referred to as “^133^Ba stock solution”) was retrieved. The specific activity of this ^133^Ba stock solution was deduced by comparison with a certified ^133^Ba point-like reference source of known activity (^133^Ba point-source from PTB, reference n° PTB-6.11-274/26.2015, calibration mark n° PTB-6.11-2006-1314, certified activity of (102.2 ± 0.55) kBq @01.01.2015 00:00:00 MEZ, uncertainty with k = 2). The activity of the ^133^Ba point-like reference source @08.06.2020 00:00:00 MEZ was calculated to be (71.48 ± 0.39) kBq, using the half-life for ^133^Ba of (3849.28 ± 2.19) d, as given in the JEFF 3.3 library. The uncertainty in the calculated activity is a combined standard uncertainty that includes the 0.057% uncertainty contribution of the half-life of ^133^Ba. The γ-spectrum of the certified ^133^Ba point-like reference source, at a sample / detector endcap distance of 8 cm, is shown in Figure S3. For the preparation of the ^133^Ba drop-source, an amount of (15.00 ± 0.05) mg of the ^133^Ba stock solution was drop-deposited on a Polyethylene (PE) foil, identical to the ones used to manufacture point-like reference sources at PTB. The PE foil had a thickness of 0.23 mm and a mass per unit area of (21.3 ± 1.8) mg·cm^‑2^. Similarly to the procedure used at PTB to produce point-like reference sources, the drop was dried on a heating plate kept at 70°C, and its activity was successively measured by γ-spectroscopy (see Figure S3). The γ-spectrum of the background is included in Figure S3. As listed in Table S7, the activity of the ^133^Ba drop-source was deduced as (26.99 ± 0.33) Bq. Therefore, a specific activity of (1799 ± 23) Bq∙g^-1^ for the ^133^Ba stock solution was deduced.

**Figure S3.** γ-spectra of the certified PTB ^133^Ba point-like reference source (in black), of the ^133^Ba drop-source (in red), and of the background (in blue). The histogram bin size is 0.36 keV. All measurements were performed at a sample / detector endcap distance of 8 cm. The reference peak of ^133^Ba used for the calibration is indicated.

**Table S7.** Area of the peak (Area) at 80.99 keV, corresponding to the γ-measurements of a ^133^Ba PTB calibrated point-like reference source and a ^133^Ba drop-source deposited on PE foil. For each measurement, the real time of measurement (t_real_) and the life time of measurement (t_life_) is reported. The calculated count rate is given as well. The activity of the PTB point-like reference source, updated to the date of measurement (i.e., 08.06.2020) is given. The calculated activity of the drop-source is given. Both γ-spectroscopy measurements were conducted at a distance between the source and the detector endcap of 8 cm.

| **^133^Ba**  **source** | **t_real_**  **(s)** | **t_life_**  **(s)** | **Area**  **(counts)** | **Count rate**  **(counts∙s^-1^)** | **Activity**  **(Bq)** |
| --- | --- | --- | --- | --- | --- |
| **PTB** | 12043 | 11200 | 4048148 ± 2036 | 361.44 ± 0.18 | (71.48 ± 0.38)∙10^3^ |
| **drop** | 86426 | 86400 | 11807 ± 117 | 0.1367 ± 0.0014 | 26.99 ± 0.33 |

**^133^Ba measurement in Position A.** For the measurements in Position A, two different samples (namely, S1 and S2) were produced from aliquots of the ^133^Ba stock solution.

*S1:* An amount of (0.41540 ± 0.00005) g of ^133^Ba in 1 M HNO_3_ was added to the PEEK vial. The activity of the added liquid source was (747.5 ± 9.4) Bq @21.07.2020.

*S2:* An amount of (0.42300 ± 0.00005) g of ^133^Ba in 1 M HNO_3_ was added to the PEEK vial. The activity of the added liquid source was (761.2 ± 9.6) Bq @21.07.2020.

Details on the γ-measurements for Position A are compiled in Table S8. γ-spectra for S1 and S2 are shown in Figure S4.

**Table S8.** Area of the peak (Area) at 80.99 keV, the real time of measurement (t_real_) and the life time of measurement (t_life_) corresponding to the γ-measurements of samples S1 and S2 in Position A. The count rate and the activity at the date of measurement (i.e., 21.07.2020) is indicated. The calculated ratio (Ratio) of the count rate of each sample over its corresponding activity is given. Both γ-spectroscopy measurements were conducted at a distance between the source and the detector endcap of 1.8 cm.

|  | **t_real_**  **(s)** | **t_life_**  **(s)** | **Area**  **(counts)** | **Count rate**  **(counts∙s^-1^)** | **Activity**  **(Bq)** | **Ratio × 100**  **(counts∙s^-1^∙Bq^-1^)** |
| --- | --- | --- | --- | --- | --- | --- |
| **S1** | 603 | 600 | 15445 ± 126 | 25.74 ± 0.21 | 747.5 ± 9.4 | 3.444 ± 0.068 |
| **S2** | 603 | 600 | 15624 ± 126 | 26.04 ± 0.21 | 761.2 ± 9.6 | 3.421 ± 0.067 |

**Figure S4.** γ-spectra of ^133^Ba used for the measurements in Position A. The histogram bin size is 0.36 keV. The nominal activity of ^133^Ba for S1 (black line) amounted to (747.5 ± 9.4) Bq. For S2 (red line), the nominal activity of ^133^Ba amounted to (761.2 ± 9.6) Bq. Both γ-spectroscopy measurements were conducted at a distance between the source and the detector endcap of 1.8 cm. The reference peak used for the activity calculations is indicated.

**^133^Ba measurement in Position B.** For the measurements in Position B, one single point-like sample was produced from an aliquot of the ^133^Ba stock solution. γ-spectroscopic measurement of the same sample was performed twice (namely, M1 and M2). An amount of (15.30 ± 0.05) mg of ^133^Ba in 1 M HNO_3_ was drop-deposited onto a 75 µm thick graphite foil. The activity of the added liquid source was (27.56 ± 0.36) Bq @15.07.2020. Details on the γ-measurements for Position B are compiled in Table S9. γ-spectra for measurements M1 and M2 are shown in Figure S5.

**Table S9.** Area of the peak (Area) at 80.99 keV, the real time (t_real_) and the life time (t_life_) of measurement corresponding to the γ-measurements M1 and M2 in Position B. Both γ-spectroscopy measurements were conducted at a distance between the source and the detector endcap of 1.8 cm. The count rate and the activity at the date of measurement (i.e., 15.07.2020) is indicated. The calculated ratio (Ratio) of the count rate of each sample over its corresponding activity is given.

|  | **t_real_**  **(s)** | **t_life_**  **(s)** | **Area**  **(counts)** | **Count rate**  **(counts∙s^-1^)** | **Activity**  **(Bq)** | **Ratio × 100**  **(counts∙s^-1^∙Bq^-1^)** |
| --- | --- | --- | --- | --- | --- | --- |
| M1 | 14406 | 14400 | 13393 ± 118 | 0.9301 ± 0.0082 | 27.56 ± 0.36 | 3.374 ± 0.069 |
| M2 | 14406 | 14400 | 13342 ± 118 | 0.9265 ± 0.0082 | 27.56 ± 0.36 | 3.362 ± 0.069 |

**Figure S5.** γ-spectra of ^133^Ba used for the measurements in Position B. The histogram bin size is 0.36 keV. The activity of ^133^Ba for both measurements M1 (black line) and M2 (red line) amounted to about 28 Bq (see Table S9). Both γ-spectroscopy measurements were conducted at a distance between the source and the detector endcap of 1.8 cm. The reference peak used for the activity calculations is indicated.

The pure geometric efficiencies of the point-like sample (with a diameter of 2.5 mm) and the electrodeposited sample (with a diameter of 20.0 mm) both placed in Position B and treated as isotropically emitting disks coaxial with a parallel circular detector were evaluated using Equation (20) of [Nuclear Instruments and Methods in Physics Research A 562 (2006) 146–153] to be 23.5768% and 22.9983%, respectively. The derived ratio *R* of 0.975 allows for transforming the count rate of a single point-like source of 2.5 mm of diameter to the count rate of a source having 20 mm of diameter. The normalized count rates (i.e., the ratio of the count rate of each sample over its corresponding activity) for S1, S2, M1, and M2 are given in Table S10. The transformed normalized count rate for M1 and M2 are indicated. From the averaged ratio of the transformed normalized count rates of the ^133^Ba samples in Position A and Position B, a geometry conversion factor of (1.045 ± 0.015) was deduced (see Table S10). This geometry conversion factor allows for a conversion of the count rate of an electrodeposited sample measured in Position B to the count rate of a volumetric sample measured in Position A. Therefore, in the calculation of the deposition yield, the count rate measured in Position B has to be multiplied by the conversion factor of (1.045 ± 0.015).

**Table S10.** ^133^Ba count rates for samples S1 and S2 (measured in Position A), and measurements M1 and M2 (measured in Position B), normalized to their respective activities. The normalized count rates for M1 and M2 multiplied by *R* = 0.975 are indicated in the column “Transformed normalized count rate”. This allows to compare the count rates of M1 and M2 to the count rates of S1 and S2. The energy independent ratios of the measurements in Position A *vs.* Position B are given. The averaged value is the geometry conversion factor to be applied in the calculation of the deposition yield.

|  | **Normalized count rate** | **Transformed normalized count rate** |  | **Ratio** |
| --- | --- | --- | --- | --- |
| **S1** | 0.0344 ± 0.0007 | **---** | **S1/M1** | 1.046 ± 0.030 |
| **S2** | 0.0342 ± 0.0007 | --- | **S1/M2** | 1.050 ± 0.030 |
| **M1** | 0.0337 ± 0.0007 | 0.0329 ± 0.0007 | **S2/M1** | 1.039 ± 0.029 |
| **M2** | 0.0336 ± 0.0007 | 0.0328 ± 0.0007 | **S2/M2** | 1.043 ± 0.030 |
|  |  |  | **Average** | 1.045 ± 0.015 |

***2.3 Gravimetrical determinations for molecular plating***

**Table S11.** Gravimetrically determined aliquot (in grams) of Dy taken from the Dy master solution and used for molecular plating. The amount of the Dy aliquot was calculated as the difference between gross weight (Dy aliquot + vial) and tare (vial). The mean and standard deviation (SD) of five consecutive weightings is indicated.

| **Weighting**  **#** | **Vial**  **(tare, g)** | **Dy aliquot + vial (gross weight, g)** | **Dy aliquot**  **(net weight, g)** |
| --- | --- | --- | --- |
| **1** | 6.79404 | 9.56813 | 2.77409 |
| **2** | 6.79409 | 9.56814 | 2.77405 |
| **3** | 6.79402 | 9.56813 | 2.77411 |
| **4** | 6.79403 | 9.56816 | 2.77413 |
| **5** | 6.79403 | 9.56814 | 2.77411 |
| **Mean** | 6.79404 | 9.56814 | 2.77410 |
| **SD** | 2.8E-05 | 1.2E-05 | 3.0E-05 |

***2.4 ^159^Dy γ-activity measurements***

The γ-spectra of ^159^Dy before molecular plating (Position A), and ^159^Dy deposited on the graphite foil (Position B) are shown in Figure S6. For sake of completeness, the background spectrum is shown as well.

**Figure S6.** Black line: γ-spectrum of ^159^Dy before molecular plating (Position A); red line: γ-spectrum of ^159^Dy deposited on the graphite foil (Position B); blue line: background. The energy bin is 0.36 keV. The reference peak used to calculate the efficiency of the molecular plating is indicated.

**3. α-spectroscopy measurements**

α-spectroscopy measurements were performed by counting at a defined solid angle. Activity measurements adopting this method, in which only the α-particles emitted into the solid angle subtended by the detector are measured, show up to 99.99% accuracy [Pommé S. Metrologia. 2015; 52(3):S73]. Figure S7 shows the α-spectrum of the ^154^Dy sample together with the ^148^Gd contamination. In the same Figure, the ^241^Am measurement using the PTB reference source recorded with a PIPS detector (A-450-21AM, Canberra; Detector sensitive area 450 mm^2^, SDD = 10.4 mm, nominal FWHM = 21 keV, counting time = 500 seconds) is superimposed.

**Figure S7.** α-spectrum of the ^154^Dy sample with the ^148^Gd contamination (blue line), and the ^241^Am PTB reference source (red line). The energy bin corresponds to 5.925 keV.

The α-peaks at 2.87 MeV and 3.18 MeV have a FWHM of 24.73 keV and 24.92 keV, respectively. These values, calculated by the Genie^TM^ 2000 Alpha Analysis Software, are close to the 21 keV nominal resolution of the used PIPS detector specified by CANBERRA. This indicates a thin deposition layer of the ^154^Dy sample. Peak-fits of the recorded α-spectrum were performed with the Origin 2021b NLFit tool (© OriginLab Corporation) by applying the following system of equations (Eq. S2 – Eq. S8), as suggested by Pomme and Marroyo in [Applied Radiation and Isotopes 96 (2015) 148 – 153] to parametrize the α-peak shape *y*_p_ for each ^154^Dy and ^148^Gd peak:

$$\left\{ \begin{aligned} \begin{aligned} z_{1}= \frac{1}{\sqrt{2}}\cdot\left( \frac{x-x_{c}}{w}+\frac{w}{t_{1}} \right) \\ z_{2}=\frac{1}{\sqrt{2}}\cdot\left( \frac{x-x_{c}}{w}+\frac{w}{t_{2}} \right) \end{aligned} \\ z_{3}=\frac{1}{\sqrt{2}}\cdot\left( \frac{x-x_{c}}{w}+\frac{w}{t_{3}} \right) \\ y_{1}=\frac{erfc(z_{1})\cdot A\cdot K}{2\cdot t_{1}\cdot(1+K+L)}\cdot e^{\left[ \frac{1}{2}\left( w/{t_{1}} \right)^{2}+\frac{\left( x-x_{c} \right)}{t_{1}} \right]} \\ y_{2}=\frac{erfc(z_{2})\cdot A}{2\cdot t_{2}\cdot(1+K+L)}\cdot e^{\left[ \frac{1}{2}\left( w/{t_{2}} \right)^{2}+\frac{\left( x-x_{c} \right)}{t_{2}} \right]} \\ y_{1}=\frac{erfc(z_{3})\cdot A\cdot L}{2\cdot t_{3}\cdot(1+K+L)}\cdot e^{\left[ \frac{1}{2}\left( w/{t_{3}} \right)^{2}+\frac{\left( x-x_{c} \right)}{t_{3}} \right]} \\ y_{P}=y_{1}+y_{2}+y_{3} \end{aligned} \right.$$

Here, *x_c_* denotes the peak center, *A* the fitted count rate area, *w* the Gaussian width component, and *t_1_*, *t_2_*, and *t_3_* the three independent low-energy tailing parameters, with *L* and *K* the relative intensities of the first and third tailing with respect to the second one. The function $erfc\left( z \right)$, defined in the Origin 2021b NLFit software as$erfc\left( z \right)= \frac{1}{\sqrt{\pi}}\int_{z}^{\infty} e^{-\frac{t^{2}}{2}}dt$, denote the Gauss complementary error functions. The entire fitted spectrum *y* is composed by 1) the two *y*_p_  peak areas of the ^154^Dy and ^148^Gd α-activity, respectively, *y*_p(Dy)_ and *y*_p(Gd)_; 2) the area contribution *y*_E_ of the low-energetic electronic noise having an exponential factor *η* and an amplitude *a*_E_; and 3) a constant background *y*_0_. The mathematical expression for *y* is given in the following system of equations (Eq. S9 and Eq. S10):

$\begin{aligned} y_{E}=\frac{a_{E}}{\eta}\cdot e^{-\frac{x}{\eta}} \\ y={y_{0}+y_{E}+y}_{P(Dy)}+y_{P(Gd)} \end{aligned}$

The fitted parameters, i.e., the optimized parameters that allow for calculating at each energy bin the corresponding *y* that best reproduce the experimental α-spectrum, are indicated in Table S12.

**Table S12.** Parameters obtained with the Origin 2021b NLFit tool for the fit of the α-spectrum plotted in Figure 3 (only uncertainties from the fitting procedure are given).

|  | **^154^Dy fit-peak** | | **^148^Gd fit-peak** |
| --- | --- | --- | --- |
| ***A*** | (0.18562 ± 0.00217) counts∙s^-1^ × keV | | (0.11885 ± 0.00145) counts∙s^-1^ × keV |
| ***x_c_*** | (2879.92 ± 0.04) keV | | (3192.9 ± 0.05) keV |
|  | | shared used peak shape parameters | |
| ***w*** | | (6.891 ± 0.040) keV | |
| ***t_1_*** | | (11.87 ± 0.11) keV | |
| ***t_2_*** | | (66.5 ± 6.5) keV | |
| ***t_3_*** | | (496 ± 172) keV | |
| ***K*** | | (4.97 ± 0.27) | |
| ***L*** | | (0.448 ± 0.077) | |
|  | | noise and background parameters | |
| ***y_0_*** | | (0 ± 1.5E-6) counts∙s^-1^ | |
| **η** | | (235 ± 142) keV | |
| **a_E_** | | (0.32 ± 0.77) counts∙s^-1^ × keV | |
| **R-Square (COD)** | | 0.99959 | |

By taking into account that a single channel represents an energy range of 5.925 keV, it is possible to obtain for each peak the count rate area *C*. Final results of the count rate in the two ^154^Dy and ^148^Gd peaks are reported in Table S13.

**Table S13.** Count rate area (*A*) of the ^154^Dy and ^148^Gd peaks for the histogram plotting the count rate per energy bin (in keV). The count rate area (*C*) of the ^154^Dy and ^148^Gd peaks for a histogram plotting the count rate per channel is reported. The uncertainties on the parameter *A* are derived from the fitting procedure. For the parameter *C*, the combined uncertainties of the fit and the Poisson counting statistics are given.

|  | ***A***  **(counts∙s^-1^ × keV)** | ***C***  **(counts∙s^-1^)** |
| --- | --- | --- |
| **^154^Dy** | 0.18562 ± 0.00217 | 0.03133 ± 0.00044 |
| **^148^Gd** | 0.11885 ± 0.00145 | 0.02006 ± 0.00032 |

It follows that the count rate for the decay of ^154^Dy is (0.03133 ± 0.00044) counts∙s^-1^. By correlating the count rate of the ^154^Dy peak to the count rate associated to the peak of the standard ^241^Am calibrated source (PTB, calibration reference n° PTB-6.11-2016-1769), an activity of (0.2126 ± 0.0040) Bq for ^154^Dy was deduced (see Table 4).
